# A Factor in Tooth-Preservation

**Published:** 1885-11

**Authors:** C. N. Pierce

**Affiliations:** Philadelphia, Pa.


ARTICLE III.

### A FACTOR IN TOOTH-PRESERVATION.

BY C. N. PIERCE, D. D. S., PHILADELPHIA, PA.

[An Address before the New Jersey State Dental Society, July 16, '85.]

*Mr. President and Gentleman of the New Jersey State Dental Society:*

I fear that I am trespassing a little upon your good nature in coming before you without a written essay, but my time has been so limited in preparing for this occasion that the very best I could do was to arrange some thoughts and give them to you as well as practicable under the circumstances. In answer to an inquiry from my friend, Dr. Palmer, as to the title of my subject,I wrote him that it would be a "Factor in Dental Caries," but I subsequently changed it to the more intelligible one announced by your president to-day, "A Factor in Tooth-Preservation;" and I am under obligations to my friend, Dr. Atkinson, for opening the way for me last evening, by his opportune remarks on the prophylactic influence of function,because that has been really the subject of my thoughts for the last three months, and was the point I endeavored to make in my remarks a month ago on the comparative anatomy of the teeth before the New England and Connecticut Vally Dental Associations at Worcester, Mass. What I shall endeavor to do now is simply to elaborate the remarks of Dr. Atkinson last evening, and confirm them with some illustrations of developement of the teeth of the lower animals, hoping with these to leave an impression upon your minds which will not soon be erased.

First, a word or two upon dental caries. If you ask the numerous teachers in this country to formulate an answer to the inquiry, What is dental caries? they will pro bably tell you that it is molecular death and disintegration of the tooth-tissue. We will not stop now to discuss the correctness of this answer. It is one that has been almost universally given to classes in dental schools, as well as in the meetings of dental societies. Molecular disintegration we have, but that this is preceded by death is doubtful indeed, and this one point in this pathological phenomenon is well worthy of consideration by any dental society. Many theories have been advanced by thoughtful men regarding the cause of this pathological condition designated dental caries. First, it was held that it was wholly due to chemical action; and there are men to day who take the ground that that is the only cause. They claim that there is some solvent (an acid) in the mouth which comes in contact with the tissues of the teeth, breaking up the continuity of the structures and dissolving out the lime-salts or inorganic portion. Then, there are others who take the other extreme, and assert that dental caries is the result of vital action; that through some deficiency in nutrition and other abnormal systemic conditions there is a loss of continuity between the hard or inorganic and the soft or organic structures, and in consequence of that loss of continuity the dissolution of the teeth naturally follows. Another theory is that the cause of decay is chemico-vital; that perverted or imperfect nutrition during the calcification of the hard tissues results in abnormality, both as to quantity and quality, this being a predisposing cause of caries, the teeth then becoming an easy prey of some solvent in the mouth, which is assumed to be an acid. We have, also, within the last few years, had advanced by our friends abroad as well as at home what is termed the parasitic theory —that decay is produced by certain low forms of vegetable or animal organism in the mouth, some of which, by their roots or mycelium, burrow into the tissues of the teeth and leave them in a condition to readily break down, and that other organisms, by virtue of their contact with the oxygen of the atmosphere, eliminate an acid, and in that way we have a solvent produced by these which disintegrates the tooth-structure. These latter theories entirely overlook the fact that many of these organisims ar merely messmates; that they live in the mouth by virtue of the pabulum on which we also live, and are not parasites at all—living on the remains of our food, on dead and refuse material, and not interfering with the live tissues in any way. Then we have still another theory, that advanced byDr. Bridgman in England, called the electrical theory; that it is by reason of a want of correspondence in the electrical conditions of the organic and the inorganic structures that the teeth are broken down.

The fault I find with all of these theories is, not that they, or most of them, have not some grain of truth in them sufficient to warrant their advancement as elements in the problem of decay, but that it is claimed by their several and special advocates that they are *the* element. In attributing dental caries to any one of these supposed causes, we seem to entirely ignore the laws governing the development and nutrition of structures.

When a tooth is developed, it is in accordance or in correspondence with law, like other tissues. Its morphology, its structural arrangement, its density, its size, its location, all are subservient to its function and nutrition. If function is delegated to some other part or organ, nutrition is likewise diverted. Health and normality in any and every respect must be preceded by normal or natural exercise of function. The arrangement of the tissues, the size, shape, and density of the teeth are not matters of whim or accident,but are due to the natural results of the mechanical forces that have been brought to bear upon them; they are the result of the degree and direction of force that has been exerted upon them by the food habit through many successive generations; they are in exact correspondence with the amount and direction of force that has been and is exercised in the preparation of the food, or in the exact ratio of the amount of resistance offered by the trituration of the food upon which the animal lives. This brings tooth-formation down to the single point of food habit, and in my estimation *tooth-formation and nutrition are the result of food habit.*

In the treatment of the mouths of many children we see unmistakable evidence of this absence of function. We risk nothing in saying to the mother or guardian of many of these patients that the food is washed into the stomach with one of various liquids without mastication, and we may with safety and great propriety add that, unless there is some change in the food habit of this child, our success in the direction of tooth-preservation will be but limited. Fluids must be restricted at meal time. Solid food must be substituted for the semi-solid, and the eight or ten minutes usually occupied in the consumption of a meal must be extended to twenty-five or thirty minutes. I say constantly to the parents of my young patients: If you want to save this child's teeth, you must banish drink from the table during meal time; let the children drink all they want before and after meals, but at meals the food should be taken as nearly dry as possible, and let the child spend half an hour or more in its mastication, utilizing the natural secretions; not washing down its food with copious draughts without an effort on the part of the teeth to triturate and prepare it for the subsequent digestive process. I bring up this point here because I want to make it more clear that, in my estimation, the loss of function is one great cause of this rapid decay of the teeth. The healthy or normal developement of the teeth is exactly in proportion to the stimulus of the resistance that is offered to them in the cutting or mastication of food.

Now, gentleman, a recognization of the foregoing is what interests *us* as dentists, and in behalf of our *patrons* or *patients* for their own welfare and comfort. In continuation of my remarks, I hope, with the aid of these specimens upon the table before us, to show you how these various tooth-forms have been the result of jaw movements, again, a necessity by virtue of the kind of food; and so in regular succession, we can safely say first, that food habit has been the important factor and controlling influence in shaping tooth-forms: second, that the restriction and limitation of diet has contributed to specialization of the teeth, third, the degree in which teeth are changed or modified in form and structure is in proportion to the differences in the degree of resistance to be overcome in the mastication of food.

For the sake of perspicuity, and at the risk of being tedious, let us first define a tooth, with its location, function, etc.

The definition given is, that it is a hard substance projecting from the surface of the mucous membrane; it is differentiated from the surrounding structures. and opposes another tooth, or a dental plate, or else in its function works against some other substance less dense. It is located in the anterior or pre-assimilative portion of the alimentary canal, and in the mammalia it is confined to the inferior and superior maxillæ, always working in a vertical or modified vertical direction, and against other teeth or some dense substance, so as to stimulate its nutrition and health.

All teeth may be arranged into five classes. First, the simple cone-shaped tooth which is represented in the cuspid of the carnivora, the prehensile teeth of all animals swallowing their prey whole, and a large class of fishes, as well as the poison-fang of reptiles and the teeth of the sperm-whale. These are among the simplest forms of teeth found in the animal economy. The next would be a chisel-shaped tooth, examples of which we see in the incisors of the rodents and other vertebrate animals. In the third class we place the trenchant-shaped teeth seen in carnivorous animals, which shut over each other like the blades of a pair of scissors, and are for lacerating or tearing. Then come the teeth which we find in the monkey tribe, having little tubercles on the triturating surface for crushing. The fifth and last class are the molars, represented by those of the elephant and of the rodents, but the most specialized or typical are those found in the herbivora, used for grinding grass and dry food. Nearly all the teeth of the animal kingdom may be placed in one of these five classes, by a little addition or subtraction corresponding with modifications in food habit and mandibular or jaw movement.

When we pick up a mandible that is armed with cone-shaped teeth, we know very well that its movement is limited to a vertical or up-and-down motion. The teeth in it are not for the trituration of food, but for seizing it. Corresponding with this cone-shaped tooth and the vertical motion which is found in all carnivorous animals, and which is not a mere matter of taste or accident, but of necessity, because of the class of food upon which the animal subsists, we find the shape of the condyle and the glenoid cavity to correspond—the latter hugging or so adapted to the former as to preclude any other motion. So we see that the food habit controls, not only the movements of the jaw and shape of the teeth, but the form and adaptation of the condyle and glenoid cavity.

We now take the other extreme in shape, represented by the molars of the rodents and the elephant. We find instead of the glenoid cavity a convex surface and the condyle a flat or slightly concave surface, which slides over the convex surface of the glenoid cavity; and this arrangement permits not only a lateral motion of the jaw, but the antero posterior which is so essential to the rodent. But the food habit of the animal was the first factor or neccessity which produced the lateral and antero-posterior motions, and these motions gave us the tooth-form, the condylar articulation of necessity following. We might follow this up through the whole anatomical structure of various animal, and find corresponding results in the digestive organs as well as in the modes of progression of the animals.

The teeth of the mammalia, and indeed nearly all of the vertebrata, are made up of three tissues—dentine, cement and enamel, the enamel-germs being present in all. In a large class of animals, as in man, these tissues are arranged with the dentine in the center, the enamel covering the dentine of the crown, and the cement covering the dentine of the root. This is the common arrangement in the teeth of all carnivorous and omnivorous animals; and in these animals we find the teeth less specialized than in the herbivora and rodentia, where, instead of having the enamel covering the crown' it is arranged in transverse lines running across the triturating surface, or the peculiar W-shaped pattern, by a dipping in of the enamel from the sides, as is seen in many of the herbivora. Where there is an antero-posterior motion of the jaw in connection with the lateral, we have these lines running transversely across the teeth, and with this the most complex structural condition. The object of this arrangement is patent to every one—the three tissues being of different degrees of density, and standing side by side, there will always be an uneven surface, with the most dense tissue prominent, which is most efficient in the preparation of the dry food upon which the animals subsists.

Again we recognize that this peculiar adaptation of the teeth to the necessities of the animal is the result of food habit. There is no exception to this rule. It is the force exercised upon the teeth which modifies their form and structural arrangement.

If you will bear with me a few moments, I will show you how true this is throughout the animal kingdom. Taking, first, some illustrations from the invertebrata—animals without a back-bone—their teeth are with few exceptions not dense, but shaped by food habit and jaw movement so as to be efficient in mastication. Commencing with this little animal which I hold in my hand, and with which we are all familiar, the echinus, designated Aristotle's lantern, because first described by him, we find that it has five teeth and five jaws, moved by thirty-five muscles. It subsists upon shell-fish, and by the movement of these teeth, with sharp, cutting edges, it drills a hole in the shell of its prey and sucks out the juices. The echinus is an animal with primitive nervous organization, yet it has sense enough to have good taste, and by its liking for shell-fish does considerable injury to the business of the oysterman. This is one of the most complex arrangements of tooth-structure that is found in the animal economy.

Our next illustration we take from the common leech. We are all familiar with the manner in which this articulate makes its wound. The animal has three jaws, which are simple semi-circles, and are armed with teeth or denticles, not for mastication, but for cutting the flesh of its prey, and making a wound from which the animal draws the blood upon which it lives. It shows the adaptation of teeth to the neccessities of the animal. The drawing upon the blackboard shows the jaws attached to the second segment, and so arrange as to make a tri-radiate wound. Among the intestinal worms, I may instance the tape-worm. You all know how difficult it is to dislodge this disgusting parasite from the alimentary canal. It has a circular mouth, armed with little hooks, which seize hold of the walls of the alimentary canal, and hold fast while the animal sucks the juices upon which it subsists. In that way these hook-shaped teeth aid the animal in obtaining its nutrition.

Then we come to the mollusks, of which the varieties described may be numbered by the thousands. We may divide them into two classes, those with and those without heads. The headless ones have, of course, no teeth; while the food habits of some with heads are without the necessity for teeth, and hence they are edentulous. But in those that have teeth we find the variety in shapes corresponding with the difference in diet; so, as the little mollusk lives upon vegetable, animal, or liquid food, the teeth quite as readily correspond to its necessities as do those of the vertebrate series to theirs. So in these, again, we have this selective influence of function, giving us structures in these plastic animals which are as fully adapted to their needs as are those enjoyed by the higher animals—*teeth modified in shape substance, and arrangement by food habit.* The different materials upon which the teeth are required to act and the different movements of the tissues in which they are implanted tend to produce that peculiar shape and structure which is most efficient for their nutrition.

Passing to the vertebrata, we have a large class of vertebrata animals whose teeth we know have been either modified or wholly lost by reason of changed food habits. Birds to-day have no teeth, yet Professor Marsh, of Boston, has described some fossil birds which were furnished with well developed teeth like those of other vertebrates. There is an immense variety of fishes, which are placed by Professor Marsh in five great classes: the leptocardia, marsipobranchii, elasmobranchii, ganoidei, and teleostei. The first of these, described by Haeckel as the acrania (without a skull), have no teeth, while the others have almost an endless variety. The marsipobranchii, of which the lamprey are examples, having pointed, horny teeth. The elasmobranchii, embracing the rays, saw-fish, sharks, etc., have teeth with sharp points peculiarly adapted to their habits of life; and so on throughout the whole series, furnishing a greater variety of tooth-formation and attachment than any other class of animals.

Before leaving the fishes I want to direct your attention to this little toad-fish which I hold in my hand. We find the body covered with spines, and a similar one in each jaw, except that their location has given them a different function, and they have become modified by virtue of it. This is an illustration of the dermal origin of the teeth, and is equally well shown by a newly-hatched dog-fish, where at this age you can scarcely distinguish the spines located on the jaw from those on the dermal surface. These, becoming modified by function, soon present a different appearance.

Next we come to the reptilia. They have but few teeth. A poison-fang is remarkable for the peculiar arrangement for conducting the poison into the wound made by it. It would be much like taking an ordinary tooth, with the enamel and dentine on it, and rolling it out flat and doubling it upon itself, the pulp cavity occupying its normal position. In folding it over we get a semi-canal connected with the sack of poison-fluid at the end of the root. The direction of the tooth is horizontal when at rest, but when elevated to pierce the prey a membrane is drawn over this semi-tube, so that it makes a complete canal, and as the animal strikes its prey the pressure upon the sac at the root ejects the fluid through the canal into the wound made by the fang. Another peculiarity is that we have an endless succession of these fang-germs, so that when one is lost another is devoloped in its place. This is true of nearly all the fish series—where teeth are lost by violence or injured by wear, new teeth soon take their place.

I have here a peculiar specimen, which represents the edentata or insectivorous animals, an ant-eater, which is deficient in front teeth. The molars it has are little round pegs, made of dentine without enamel. The front teeth are deficient, yet in some of this group there is a lateral incisor, and in nearly all there are germs of both lateral and central incisors. They have not been developed for generations, yet the germ being present, represents the original idea and form of development, although it is aborted. Loss of function has greatly modified the teeth of this animal; the relegation to the tongue of the function of the incisors has made those teeth no longer necessary; hence they have disappeared, only the germs remaining to indicate the former type. The posterior teeth, having no hard substances to grind, have wholly lost their enamel; they are specialized for the services of the animal. This is not the true armadillo, although allied to that family.

As teeth are specialized by function and adapted to certain kinds of food, they are usually reduced in number; so, also, as we go up in the scale of intelligence from the lower to the higher, increased brain development seems to have a similiar influence, the ancestral animal usually having had a greater number. Relegation of function brings diversion of nutrition.

Next in order comes a class of aquatic animals, which includes the sirena, or sea-cow, an herbivorous animal living in the water, and which is furnished with molars adapted to its diet. To this class of aquatic animals belongs also the spermaceti or sperm-whale, whose teeth are strong and cone-shaped, giving us the idea of prehensile use, and ranging in size in correspondence with location in the jaw, the heavier ones being located nearer the articulation. Its prey is seized and swallowed whole.

In the mysticetus. or right whale, balænoida(the largest mammal), we find a set of teeth in embryo, but they are functionless and absorbed before birth. At birth, in place of teeth are developed thin plates that run transversely across the jaw, some two hundred in number, and varying from ten to twenty feet in length. These great plates, which furnish the whalebone of commerce, are attached to the upper jaw, and form a sort of a fringe on their lower edge, in which, as the animal swims through the water with open mouth, thousands of small, jelly-like animals which abound therein become entangled. The water being expelled, these are transferred to the œsophagus of the whale and become its food. These plates are an adaptation of teeth specialized to the needs of the animal, and serves it in its nutritional demands.

In the quadrumana, embracing the lower monkeys and lemurs, we have teeth for crushing fruits—tubercular teeth, and very closely allied to those of the human family, but somewhat different in form, and in some greater in number, the cuspids being more prominent and serving the males for weapons in combat.

Then we come to the anthropoidea, a group that embraces man as well as the higher apes. This group has teeth alike, save in the prominence of the cuspids; but in this ascent in the scale towards man we lose some of the teeth, the lemurs and lower monkeys having thirty-six, while the anthropoidea have but thirty-two. And it is a question worthy of consideration whether the frequent absence of the third molar in the human family is not in the same line of reduction; absence of function sending the nutritive current to other localities.

It is probable that, with a continuance of our present diet and manner of living, it will not be many centuries before man will have twenty-eight instead of thirty-two teeth. It is also probably that this reduction will be facilitated by the efforts of specialization, which is constant.

Man is an omnivorous animal, and in his mode of living his teeth are not subjected to the use or kind of diet which is best calculated to insure their health. If we had the opportunity of examining any large class of people who were now and had been for some centuries confined to a limited diet,with little or no animal food, we should expect to find incisors well developed, cuspids somewhat suppressed, and molars assuming a more herbivorous type, having cutting tubercles, and showing a tendency to the infolding of the enamel.

We do know that during the period in this country when the negro of the South was confined to a course diet he had fine incisors and strong molars. His cuspids were not more prominent than is seen in the higher races. This we should attribute to the fact of his diet being largely granivorous and coarse. You know that in all strictly herbivorous animals the cuspids are either entirely deficient or are merely present in a transitionary form.

The carnivorous animals, whether aquatic, terrestrial, arborial, or fusorial in their habits, or whether occupying the polar or equatorial regions, are alike true to their cuspids and carnivorous molars—illustrating again the influence of food habit.

The rodentia, of which this beaver is a very good type, have finely-developed incisors growing from permanent pulps, and molars with transverse lines of enamel. These forms are the result of the gnawing habit which necessitates the antero-posterior movement of the mandible. Accompanying this is also the peculiar arrangement of the three hard tissues of the teeth, which always gives the incisors a sharp, cutting edge, by placing the enamel, which is most dense, on the external or labial surface; the dentine next, and the softer tissue, not unlike cementum, on the internal or palatine surface.

Now, Mr. President and gentlemen, I might continue these illustrations through every modification of the animal kingdom, and show you that, whenever there is a differentiation in the food habit, there is a corresponding one in mandibular movement, which is accompanied by a tooth-formation resulting therefrom, and that the condylar attachment or articulation is so constant and true to the mandibular movement and tooth-form that, when once recognized, there would be no difficulty in describing the movement of jaw and tooth-form belonging thereto.

In recognizing the conditions which induce morphological pecularities and modifications in dental structures, we certainly have some light thrown upon a condition which might induce tooth-degeneration—in the one case functional activity, followed by healthy nutrition; in the other, functional inactivity, or the absence of function, following by diversion or relegation of nutrition to other localities.

In conclusion, gentlemen, let me once more impress upon you the importance of the influence of function as a prophylactic agent, and suggest that, in our duty to our patrons, we can render them no better service than by enlightening their understanding to this extent.—*Dental Cosmos*.

